# Unobtrusive Mattress-Based Identification of Hypertension by Integrating Classification and Association Rule Mining

**DOI:** 10.3390/s19071489

**Published:** 2019-03-27

**Authors:** Fan Liu, Xingshe Zhou, Zhu Wang, Jinli Cao, Hua Wang, Yanchun Zhang

**Affiliations:** 1 School of Computer Science, Northwestern Polytechnical University, Xi’an 710072, Shaanxi, China; zhouxs@nwpu.edu.cn (X.Z.); wangzhu@nwpu.edu.cn (Z.W.); 2 College of Engineering and Science, Victoria University, Melbourne, VIC 3011, Australia; Hua.Wang@vu.edu.au (H.W.) Yanchun.Zhang@vu.edu.au (Y.Z.); 3 Department of Computer Science and Information Technology, La Trobe University, Melbourne, VIC 3086, Australia; J.Cao@latrobe.edu.au

**Keywords:** hypertension identification, class association rule (CAR), classification, association rule mining, heart rate variability (HRV), ballistocardiogram (BCG)

## Abstract

Hypertension is one of the most common cardiovascular diseases, which will cause severe complications if not treated in a timely way. Early and accurate identification of hypertension is essential to prevent the condition from deteriorating further. As a kind of complex physiological state, hypertension is hard to characterize accurately. However, most existing hypertension identification methods usually extract features only from limited aspects such as the time-frequency domain or non-linear domain. It is difficult for them to characterize hypertension patterns comprehensively, which results in limited identification performance. Furthermore, existing methods can only determine whether the subjects suffer from hypertension, but they cannot give additional useful information about the patients’ condition. For example, their classification results cannot explain why the subjects are hypertensive, which is not conducive to further analyzing the patient’s condition. To this end, this paper proposes a novel hypertension identification method by integrating classification and association rule mining. Its core idea is to exploit the association relationship among multi-dimension features to distinguish hypertensive patients from normotensive subjects. In particular, the proposed method can not only identify hypertension accurately, but also generate a set of class association rules (CARs). The CARs are proved to be able to reflect the subject’s physiological status. Experimental results based on a real dataset indicate that the proposed method outperforms two state-of-the-art methods and three common classifiers, and achieves 84.4%, 82.5% and 85.3% in terms of accuracy, precision and recall, respectively.

## 1. Introduction

Hypertension is a common and risky cardiovascular disease in modern society, which manifests as continuously elevated arterial blood pressure. According to a report from the World Health Organization, hypertension affects more than 40% of adults aged 25 and over, which signifies that globally over one billion people suffer from hypertension [1]. Without timely treatment, it can lead to serious complications, such as strokes, heart attacks, kidney failure, and so on [1]. As a result it is reported that hypertension causes over 9.4 million deaths per year [1]. Problematically, hypertension may not show any symptoms for many years or even decades. Indeed, many people do not realize that they already suffer from hypertension. In the US, nearly 13 million patients are unaware of their condition, while in China, such patients account for about 59% of the population [1]. Therefore, identifying hypertension as early as possible is crucial to alleviate the problem and avoid more damage to the human body [2].

The cuff-based mercury sphygmomanometer is the traditional clinical method to measure blood pressure. However, it requires specialists to operate and is uncomfortable to use, which is not suitable for daily use in the home environment. More importantly, hypertension is a quite complex physiological status where blood pressure always fluctuates unpredictably. However, sphygmomanometers can only give discrete measurement results, which is not conducive to accurately analyzing the disease status. Hence, novel hypertension identification methods that can solve the above problems are needed.

Existing clinical studies showed that blood pressure is simultaneously controlled by the sympathetic nerve and parasympathetic nerve, and that heart rate is as an effective clinical metric to reflect the function of autonomic nerve system (sympathetic nerve and parasympathetic nerve are collectively called the autonomic nerve system) [3,4]. Therefore, quantitatively investigating cardiac autonomic deregulations by analyzing heart rate variability (HRV) has become a mainstream method in the field of cardiovascular research, including hypertension identification [5]. For example, Poddar et al. [6] proposed a HRV-based machine learning method to classify normal and hypertensive cases. It first extracted RR intervals sequence (a RR interval is the time interval between two successive heartbeats) from five-minute electrocardiogram (ECG) collected by using a commercial device. Then, it extracted lots of linear-nonlinear features to characterize the fluctuation pattern of HRV. Finally, a support vector machine (SVM) was trained to distinguish hypertensive patients and normotensive subjects. Ghosh et al. [7] extracted not only HRV-related features from photoplethysmograph (PPG) data, but also many other features from galvanic skin response and skin temperature to model the hypertension patterns. Afterwards, classifiers including K-Nearest Neighbors, Naïve Bayes, Decision Trees and SVM were employed to identify hypertension patients. Due to the poor quality of the signals collected by utilizing a smart watch, only an F-measure of 0.62 was obtained. Melillo et al. [8] proposed a processing pipeline for 24-h ECG Holter recordings to identify high-risk hypertensive patients. The pipeline designed plenty of HRV features, from which the most powerful ones were selected by using unpaired *t*-tests and chi-square tests. Finally, the performance of different combinations of features and machine learning algorithms were compared. Although these studies have made great progress, there are still two problems that need to be addressed. First, existing methods usually extract limited features from only one or two aspects (i.e., time-frequency domain and the non-linear domain) to model the hypertension pattern. However, as a complex physiological status, hypertension pattern is hard to accurately characterize by utilizing features extracted from limited aspects [5]. Second, existing studies usually utilize classifiers such as SVM, artificial neural network and logistic regression to identify hypertension. Although commonly used classifiers can determine whether a subject suffers from hypertension, their classification results are not interpretable. In other words, their classification results cannot give more detailed information about why subjects are hypertensive or normotensive. Consequently, these classifiers cannot assist doctors to analyze patient’s condition in depth.

Ballistocardiogram (BCG) [9] is a kind of non-intrusive physiological signal that can accurately reflect the micro body vibrations caused by the mechanical activities of heart. To be specific, BCG is defined as the reaction (displacement, velocity or acceleration) of the whole body resulting from cardiac ejection of blood. Consequently, BCG signal is a combination of multiple forces related to the movements of the heart itself, and the blood inside the heart and arteries [10,11]. Since BCG signal contains information about the heartbeats, it has been widely employed in diagnosing varieties of cardiovascular diseases, having achieved great successes [12,13]. For example, Liu et al. [12,14] put forward a novel obstructive sleep apnea detection method based on the segmentation of BCG signals. To solve the two problems of existing hypertension identification methods mentioned above, this paper proposes a novel BCG-based hypertension identification method, whose key idea is to integrate association rule mining and classification together, aiming to use the association relationship among features to model and identify hypertension pattern. Concretely, the proposed method first extracts multiple features from BCG to characterize the hypertension pattern comprehensively. Afterwards, it mines and selects a set of representative class association rules (CARs) from the extracted features to represent the association relationship among different features. Finally, based on the extracted CARS, a classifier called CAR-Classifier is constructed to discriminate hypertensive patients from normotensive subjects. In particular, the proposed method not only can obtain high hypertension classification performance, but also can generate interpretable classification results (i.e., the extracted CARs), which is conducive to analyzing patients’ condition in depth. The contributions of this work are three-fold:

- First, to characterize the hypertension pattern more comprehensively, we extract lots of features from various aspects based on BCG. To be specific, on the one hand we extract HRV-related features from time domain, frequency domain and non-linear domain to model the function of sympathetic nerve and parasympathetic nerve. On the other hand, we also design a set of four features to characterize the fluctuation pattern of BCG signal itself, so as to investigate whether the fluctuation pattern of BCG signal also has a relationship to hypertension status. Experimental results indicate that the features extracted from multiple aspects can accurately characterize the pattern of hypertension. In addition, it also shows that the BCG signal of hypertensive patients usually fluctuates more wildly than that of normotensive subjects, which shows the usefulness of the BCG fluctuation features.
- Second, in order to distinguish hypertensive patients and normotensive subjects more accurately, we construct a CAR-Classifier by integrating association rule mining and classification together. With the help of the CAR-Classifier, the association relationship among extracted features can be fully investigated and exploited, which significantly increases the classification performance. Furthermore, the CAR-Classifier can also generate a set of CARs. These CARs contain plenty of meaningful information about the patients’ physiological status, which is proved to be useful for analyzing the patients’ condition in-depth.
- Third, we conduct extensive experiments to evaluate the proposed method based on a real BCG dataset of 128 subjects. Experimental results show that the performance of the proposed method is better than two state-of-the-art methods as well as three common classifiers, obtaining 84.4%, 82.5% and 85.3% in terms of accuracy, precision and recall, respectively. In addition, we also evaluate the effectiveness of the generated CARs by carrying out a small-scale user study, and the experimental results show high utility of these CARs in diagnosing hypertension.

The rest of this paper is organized as follows: We review the related work in Section 2. Then, the materials and the methods are described in Section 3, followed by the experimental results in Section 4. Next, we discuss the proposed method in Section 5. Finally, the paper is concluded in Section 6. An early conference presentation of this work was described in [15].

## 2. Related Work

In this section, we will briefly review the related work, which can be grouped into two categories as follows:

The first category focuses on the feature extraction for hypertension identification. It has been verified that HRV has a strong relationship with hypertension [3]. Hence, HRV is usually utilized for identifying hypertension. At present, there are three commonly used HRV analysis methods, mainly including time domain analysis, frequency domain analysis and non-linear domain analysis [16]. The time domain analysis is to compute several statistics, so as to characterize the pattern of hypertension. These statistics contain plentiful information of heart, blood vessels and nervous-humoral regulation. Yue et al. [17] found that the commonly used time domain features of masked hypertensive patients are significantly decreased compared with those of healthy people, which indicated that time domain analysis could be used to discriminate between healthy people and hypertensive patients. Frequency domain analysis is usually used to investigate the power distribution of the RR intervals sequence. Aldemir et al. [18] analyzed the power in different frequency bands and found that there was a statistically significant difference between the features (for example, the ratio of the power in low frequency band to the power in high frequency band) extracted from hypertensive patients and normotensive subjects respectively. Linear HRV analysis methods assume that the RR interval sequence is stationary or that variations are harmonic or sinusoidal in nature. However, the heart rate actually is continuously modulated by non-linear functions. Therefore, non-linear analysis is necessary for HRV analysis. By analyzing the RR interval sequences of pregnant women, the work in [19] found that there was an obvious relationship between the complexity of HRV and the blood pressure. It is notable that the aforementioned works analyzed HRV from only one or two domains, which resulted in limited classification performance. In contrast, this paper extracts features from time-frequency domain and non-linear domain simultaneously. In addition, this paper also designs a set of features to model the fluctuation pattern of BCG, which are experimentally proved to be useful for identifying hypertension.

The second line of studies aim to construct machine learning-based hypertension classification models. Ni et al. [5] proposed a temporal pyramid representation and feature pooling technique-based HRV analysis approach, by which the ECG signal was subdivided into several temporal resolution levels and the HRV features extracted from different levels were then aggregated into a single feature vector. Finally, L_1_-regularized logistic regression and SVM were utilized to distinguish hypertensive patients and normotensive subjects. Poddar et al. [20] extracted a lot of HRV-related features from RR interval sequences and then directly fed them into a SVM to identify hypertensive subjects. In order to characterize hypertension pattern more comprehensively, Ghosh et al. [7] extracted features from multiple physiological signals and then modeled them by separately employing five common classifiers. Finally, the Adaboost model achieved the highest identification performance with all the extracted features utilized. Particularly, Rundo et al. [21] proposed a PPG based non-invasive cuff-less blood pressure estimation method. It collected the PPG signal of patients by employing a silicon photomultiplier sensor (SiPM), and then extracted three groups of features to characterize the correlation between the acquired PPG signal and blood pressure. The features were further modeled by two artificial neural networks separately. Eventually, an average error of two mmHg for systolic blood pressure estimation and diastolic blood pressure estimation was obtained. Although these methods achieved good performance, they can only judge whether the subjects are hypertensive, but they cannot explain why the subjects suffer from hypertension, which is unhelpful for doctors to analyze the patient’s condition in-depth. To solve this problem, Melillo et al. [8] applied a decision tree-based classifier to model the HRV-related features extracted from ECG signal, and thus obtained a set of dendroid decision rules. If a subject matched a rule’s antecedent, he or she would be classified into the corresponding class, i.e., the rule’s consequent. The obtained dendroid decision rules to a certain degree facilitate the explanation of why subjects suffer from hypertension. However, it only considered just one feature at each node when constructing the decision tree, which might lose the correlation relationship among features and hence lead to limited classification performance. To this end, this paper puts forward a CAR-Classifier to identify hypertension. It considers all the extracted features simultaneously when extracting CARs, which significantly improves the identification performance. In addition, it can also generate a set of informative CARs that can accurately reflect the subjects’ physiological state.

## 3. Materials and Methods

In this section, we first describe the process of collecting experimental BCG dataset, and then elaborate the details of the proposed method. To facilitate the reproducibility of the research, the software code and BCG dataset are published online at https://doi.org/10.6084/m9.figshare.7594433 (Supplementary Materials).

### 3.1. Collection of Real BCG Dataset

**Figure 1:**
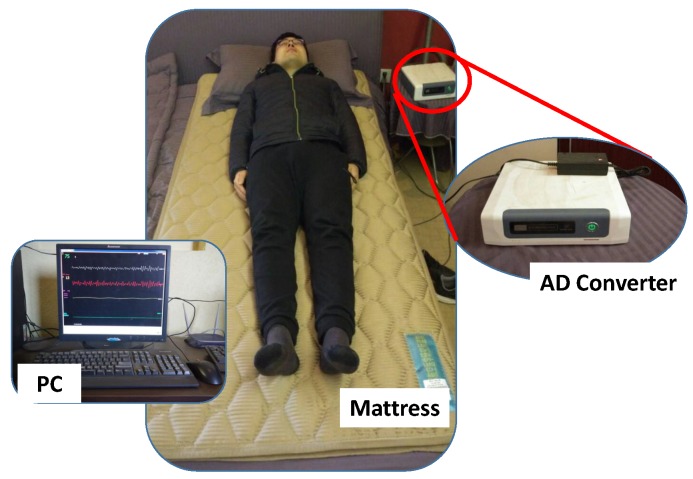
The micro-movement sensitive mattress based BCG signal acquisition system (RS-611).

To non-intrusively and conveniently collect BCG signal, an unobtrusive BCG signal acquisition system (named RS-611) is employed in this study [22]. The RS-611 is a certificated medical device developed by Institute of Air-force Aviation Medicine (Beijing, China). As shown in Figure 1, the RS-611 is comprised of a micro-movement sensitive mattress (MSM), an analog-digital (AD) converter and a terminal PC. The MSM is the main function part of this system, in which two hydraulic pressure sensors (oil tubes) in the shape of strips are embedded. Particularly, one is located at the upper part of the mattress (i.e., chest area) and the other one is placed at the leg region. Both of them are long enough and placed in parallel, which guarantees that proper BCG signal can be captured regardless of the size of the subject. When a subject lies on the MSM, the changes of original pressure caused by cardiac activities (i.e., heartbeats) are recorded, amplified, and converted to digital signal by utilizing the 16-bit-resolution AD converter with a sample rate of 100 Hz, forming a time series of composite pressure data. Afterwards, BCG signal is separated from the original composite pressure signal by performing drift compensation and digital filtering based on the built-in software, and eventually displayed on the PC. In [23], a real-time comparison between BCG signal collected by RS-611 and ECG signal collected by a commercial ECG collector (Prince 180D, http://www.healforce.com/en/) was made, whose experimental results indicated that each wave trough of the collected BCG signal corresponded to a local minimum point of the collected ECG signal, which demonstrated the suitability of the collected BCG signal for analyzing and diagnosing cardiovascular diseases. In comparison with other wearable signal acquisition devices like ECG Holter [24], smart watch [25], smart chair [26], PPG based non-invasive blood pressure estimator [27] and cuff-based mercury sphygmomanometer, the MSM-based RS-611 is completely unobtrusive and the users do not need to attach any electrodes or devices on their body. Furthermore, the RS-611 does not require complex configurations, which is especially suitable for the elderly.

In this study, 175 staff from our university were recruited as subjects. All of them gave their informed consent for inclusion before they participated in the study. The study was conducted in accordance with the Declaration of Helsinki, and the protocol was approved by the Medical Experimental Ethical Inspection Institute of Northwestern Polytechnical University (No.20170078). First, we conducted routine examinations to obtain basic physiological index. In particular, the blood pressure was measured by a trained nurse by using a standard mercury sphygmomanometer. At each examination, blood pressure was measured in the left arm twice after five minutes of rest. According to the guidelines of the American Society of Hypertension [28], the subjects were asked to be in seated position when measured. For each subject, blood pressure was recorded in four separate sessions within 3 weeks, and the averaged values were then used to represent the final systolic and diastolic blood pressure values. Subjects with blood pressure values greater than 140/90 mmHg were viewed as hypertensive patients, while the rest were regarded as normotensive subjects. In particular, subjects were excluded if they met any of the following criteria [29]: (1) use of any anti-hypertensive drugs when measuring blood pressures, (2) Body Mass Index (BMI) >30, (3) history or clinical evidence of congestive heart failure or myocardial infarction, (4) atrial fibrillation, (5) diabetes mellitus, (6) alcoholics or smokers. After applying these exclusions, 68 hypertensive patients and 76 normotensive subjects were eligible for our experiments.

Before collecting BCG signal, we instructed each subject about how to use the RS-611 properly. In addition, the day before the experiment they were asked to sleep on the bed for about one hour for the sake of gathering feedbacks. We adjusted the indoor temperature, indoor light intensity, noise level and other factors that may affect their normal sleep according to their feedbacks, aiming to make sure that can sleep well through the whole night. Finally, for each subject one night of BCG signal was collected when they were sleeping, since the physiological indexes are relatively stable and not affected by other factors during sleep. The next morning, subjects were asked some questions, for example, when did they fall asleep, when did they get up, and how did they sleep. If the subjects did not sleep well or the sleep time was less than seven hours, the corresponding BCG recordings were considered inappropriate for research and were therefore discarded. Furthermore, a manual visual inspection was conducted on each BCG recording. BCG recordings that contained too many drastic fluctuations were also excluded from this research. After the inspection process, the final BCG dataset left 128 BCG recordings in total, including 61 hypertensive patients and 67 normotensive subjects. The statistical results of the BCG dataset are summarized in Table 1.

### 3.2. Hypertension Identification Method Framework

**Figure 2:**
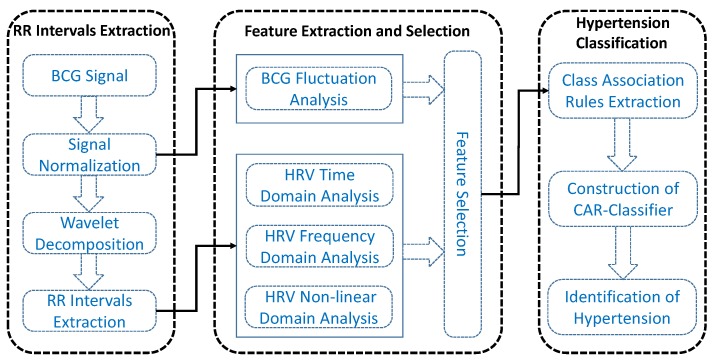
The framework of the proposed hypertension identification method.

In this paper, we aim to exploit the association relationship among multi-dimensional features to automatically distinguish hypertensive patients and normotensive subjects. As shown in Figure 2, the proposed hypertension identification method mainly consists of three stages, i.e., RR intervals extraction, feature extraction and selection, hypertension classification.

During the RR intervals extraction stage, we first normalize the BCG in order to eliminate signal amplitude differences caused by different body weights, then we utilize the multi-resolution wavelet analysis to extract the approximate layer signal that can clearly reflect the cardiac activities of heart. Finally, we compute the RR intervals sequence by using an overlapping sliding window algorithm. In the feature extraction and selection stage, we not only extract HRV-related features from time-frequency domain and non-linear domain, but also extract four BCG fluctuation-related features to characterize the fluctuation pattern of BCG. Moreover, we compute significant difference level for each feature, and select features with excellent discrimination ability for baseline methods. In the third stage, we first mine and select a set of informative CARs from the obtained features, based on which we then construct the CAR-Classifier and finally classify the subjects into hypertensive and normotensive.

### 3.3. RR intervals Sequence Extraction

**Figure 3:**
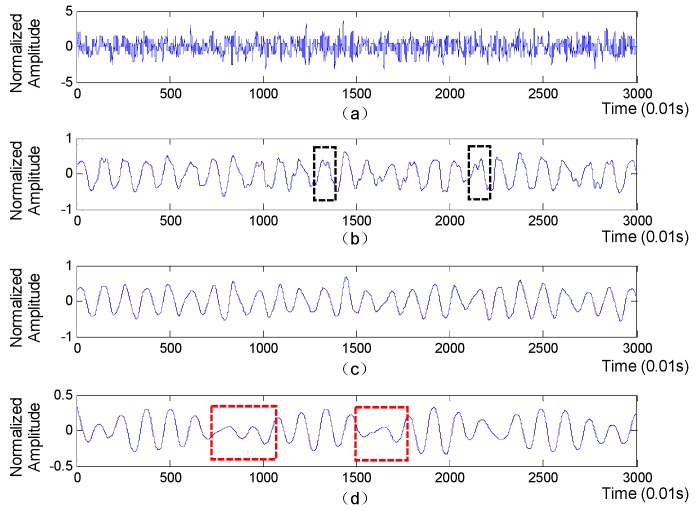
The original BCG and the approximation layers of the BCG. (**a**) The original BCG signal; (**b**) The 4th approximation layer; (**c**) The 5th approximation layer; (**d**) The 6th approximation layer.

A segment of collected BCG signal is shown in Figure 3 (top subplot), from which we will extract accurate RR intervals sequence for further extraction of HRV-related features.

The extraction of RR intervals sequence mainly consists of three steps as follows:

*Signal normalization:* Since the amplitude value of BCG signal is influenced by body weight to a certain extent, we first eliminate BCG signal amplitude difference caused by different body weight by normalizing the BCG signal using Z-score method [13], which is written as:

(1) $$X_{nor\_i}=\frac{X_{i}-\mu }{\sigma },$$

where $X_{i}$ is the $i_{th}$ value of BCG signal, μ and σ represent the mean value and the standard deviation of the BCG signal respectively, and $X_{nor\_i}$ is the normalization form of $X_{i}$. Afterwards, we design an elliptic bandpass filter to filter the noise component that is irrelative to the heart beats. Based on extensive experiments, the passband corner frequency and the stopband corner frequency are set to 5/6 Hz and 13/6 Hz respectively and the passband ripple and the stopband attenuation are set to 0.2 and 8 respectively.

*Wavelet decomposition:* Discrete wavelet transform (DWT) is a signal processing technique that is often employed to extract useful information from non-stationary time series, including physiological signals [14,15]. The utilization of DWT usually has a wonderful advantage that it can decompose the time series into multiple time-frequency resolutions, by which the details of the signal in time and frequency domains can be clearly displayed. Therefore, in this paper we utilize DWT to decompose BCG signal and further select the most appropriate approximation layer that can clearly reflect the heartbeats. Concretely, the BCG signal is first decomposed into detail component and approximation component at the first level, which is implemented by passing it through a pair of high-pass and low-pass filters as follows:

(2) $$Detail\left(n\right)=\sum_{k=-\infty }^{\infty }x_{\left(k\right)}\varphi_{h}\left(2n-k\right)$$

(3) $$Approximation\left(n\right)=\sum_{k=-\infty }^{\infty }x_{\left(k\right)}\varphi_{g}\left(2n-k\right)$$

where $x_{\left(k\right)}$ represent the *k*_th_ sample of BCG signal, and $\varphi_{h}$ and $\varphi_{l}$ are high-pass filter and low-pass filter respectively. The detail and approximation components are comprised of detailed coefficients and approximant coefficients, respectively. Afterwards, the above process is iteratively applied to the approximation component at each level until a specified level is reached. Finally, the approximation coefficients at each level are separately utilized to reconstruct an approximation layer of the original BCG signal. The approximation layers derived from BCG signal is shown in Figure 3, with the ‘db6’ function used as the wavelet basis. Obviously, compared with the 5th approximation layer, the parts in red boxes mean that the 6th approximation layer may miss some heartbeats, while the parts in black boxes indicate that the 4th approximation layer is prone to generate some fake wave peaks, which will lead to inaccurate RR intervals. Moreover, the waveform of the 5th approximation layer is more regular and smoother than that of the 4th and 5th approximation layers. Therefore, the 5th approximation layer is finally selected to extract RR intervals.

**Figure 4:**
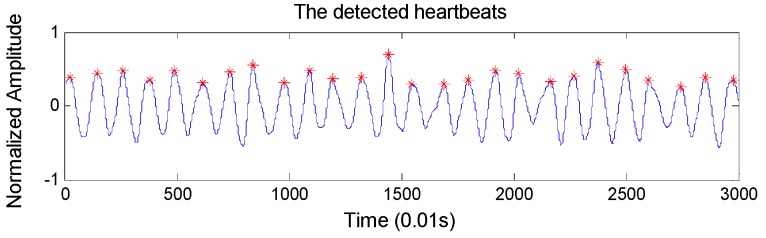
The detected heartbeats based on the 5th approximation layer of the BCG signal.

*RR intervals extraction:* To locate the heartbeats accurately, we design an overlapping sliding window to detect BCG wave peaks automatically. Concretely, the window size and the overlapping size are set to 100 samples and 60 samples respectively (the sample frequency of the BCG signal is 100 Hz), considering that the normal heart rate range is 60–100 per minute. The detected heartbeats are shown in Figure 4, which shows very high heartbeat detection performance. In particular, it achieves an accuracy of 98.4% in detecting the heartbeats compared with ECG monitor.

### 3.4. Feature Extraction and Selection

HRV can reflect the function of sympathetic nerve and parasympathetic nerve, and has being usually used for identifying hypertension [30]. On the other hand, we also notice that the fluctuation of BCG is directly caused by cardiac mechanical activities. In other words, analyzing the fluctuation pattern of BCG may be also helpful for identifying hypertension [12]. Therefore, in this section we not only extract HRV-related features from RR intervals sequence, but also extract a set of four features to model the fluctuation pattern of BCG. All the extracted features are displayed in Table 2, which can be categorized into two groups as follows.

#### 3.4.1. Heart Rate Variation (HRV) Analysis

To characterize hypertension patterns more comprehensively, we extract HRV-related features from the time domain, frequency domain and non-linear domain simultaneously. Time domain analysis can obtain rich information about blood vessels, heart and nervous-humoral regulation [4,31]. In particular, the MEAN, SDNN, RMSSD and PNN50 are extracted as time domain features, which represent the mean, the standard deviation, the root mean square of RR intervals, and the percentage of RR intervals that are longer than 50 ms, respectively. The first three features are utilized to depict the entire distribution of RR intervals, while the last feature is used to model the degree of fluctuation of the RR intervals.

Frequency domain analysis has been proved to be effective for assessing the variation of the autonomic nervous system [4]. It can effectively characterize the balance of the sympathetic nerve and parasympathetic nerve [32]. In this study, the power values in different frequency bands are extracted as frequency features by utilizing Fast Fourier Transform (FFT) technique. The extracted frequency features include vLF, LF, HF, and LF/HF. Specifically, vLF represents the power in very low frequency band ranging from 0.0033–0.04 Hz, which reflects vascular mechanisms caused by negative emotions. LF denotes the power in low frequency ranging from 0.04–0.15 Hz, which reflects sympathetic modulation of heart rate. HF means the power in high frequency band ranging from 0.15–0.4 Hz, which reflects the parasympathetic nervous system activity. LF/HF is the ratio of the power in LF and HF, which measures the balance of the sympatho-vagal balance [5]. It is notable that the RR intervals sequence is resampled by using cubic spline interpolation with a sampling rate of 4 Hz before extracting these features [33].

The extracted non-linear features include Sample Entropy (SampEn) and Detrended Fluctuation Analysis (DFA), which are two of the most useful features for studying the variability, irregularity and complexity of RR intervals sequence in existing cardiovascular diseases-related researches [5,12,19,34,35,36]. Particularly, SampEn is an effective metric for measuring the complexity and regularity of short time series [34,37]. Higher SampEn usually means that the signal is of higher complexity. Given a signal sequence, there are two important parameters when calculating the SampEn value, i.e., the detail level of vector *m* and the threshold *r* used to filter out the noise. According to the widely accepted empirical guidelines, we set the parameters as *m = 2* and r = 0.15 * STD (STD is the standard deviation), respectively. On the other hand, DFA is good at mining the long term inner correlation of time series [38]. Since BCG signal is also influenced by respiration which presents a certain periodicity and may distort the RR intervals sequence, we use DFA to eliminate the trends caused by respiration and hence focus on the inner correlation of heart beats. In DFA analysis, we introduce a parameter denoted as *s*, which represents the length of signal segments. To avoid the influence of respiration on heart rate (there are about 3–6 heart beats during one breath), the segment length is set to 40 ≤ s ≤ 240.

#### 3.4.2. BCG Fluctuation Analysis

Since the fluctuation of BCG signal is modulated by cardiac mechanical activities, it might be feasible to study cardiovascular diseases such as hypertension by analyzing the fluctuation pattern of BCG. However, the BCG signal usually contains noise caused by body movements and signal acquisition device itself, the features we will extract should be insensitive to noise. To this end, four anti-noise features that can characterize the fluctuation pattern of BCG signal from different aspects are designed as follows:

*Zero Crossing Rate (ZCR):* ZCR is an efficient feature for characterizing the signal complexity and is usually employed to detect percussive sound signal which is similar to BCG signal. Using *sgn()* to denote the sign function that returns -1 for a negative argument, 0 for 0, and 1 for a positive argument, then the ZCR of is defined as:

(4) $$ZCR=\frac{\sum_{i=2}^{N}\left|sgn\left(S_{i}\right)-sgn\left(S_{i-1}\right)\right|}{2\left(N-1\right)}$$

where $S_{i}$ denotes the amplitude value of the $i_{th}$ sample point, and *N* represents the total number of sample points of BCG signal. Intuitively, ZCR measures how many times two successive samples go through zero in a unit of length. Thus, recordings with higher ZCR values usually fluctuate more violently.

*Average Cumulative Amplitude Change (ACAC):* Intuitively, given a time series signal with fixed sample frequency, the more intensive the signal fluctuations, the larger the difference between two adjacent sample values. Therefore, we design ACAC to measure the degree of changes in amplitude, so as to model the fluctuation pattern of BCG signal. The ACAC is defined as follows:

(5) $$ACAC=\frac{\sum_{i=2}^{N}\left|S_{i}-S_{i-1}\right|}{T}$$

where $S_{i}$ denotes the amplitude value of the $i_{th}$ sample point, and T represents the corresponding time duration. Particularly, the utilization of T is to eliminate the effect caused by different length of signal.

*Average Number of Extreme Points (ANEP):* As a typical cardiovascular disease, hypertension can affects the normal heart activities to a certain degree, which can be reflected in the degree of chaos of the BCG signal. Through careful observation of the experimental dataset, we find that the BCG signals collected from hypertensive patients usually show more signal thorns compared with that collected from normotensive subjects. Moreover, we notice that the more serious the condition of hypertension is, the more signal thorns the BCG signal presents. Since signal thorns can be viewed as signal extreme points, we characterize the aforementioned phenomenon by counting the average number of extreme points, which is defined as:

(6) $$ANEP=\frac{len\left(find\left(diff\left(sgn\left(diff\left(S\right)\right)\right)==\pm 2\right)+1\right)}{T}$$

where *len()* represents the size of the array, *find()* returns elements that meet the defined condition, *diff()* denotes the derivatives of the array, and *sgn()* is basic symbol function. Intuitively, higher ANEP value usually represents more chaotic BCG signal. In order to facilitate the understanding, we explain the above function step by step as follows: *diff(S)* is the first-order derivative of the BCG signal, which is comprised of positives, negatives and zero. It is notable that the samples corresponding zero in *diff(S)* are not necessarily extreme points. Actually, only three-element arrays in the shape of (positive, 0, negative) or (negative, 0, positive) in *diff(S)* can denote extreme points. To find out these three-element arrays, we further convert *diff(S)* into *sgn(diff(S))*, which consists of number 1, number −1, and zero. Then, we compute the second-order derivative as *diff(sgn(diff(S)))*, where the three-element arrays mentioned above have been transformed into forms of (0, 2, 0) or (0, −2, 0). Next, we locate the these extreme points by using *find(diff(sgn(diff(S)))==±2)*. Finally, the extreme points in BCG signal is counted and further normalized as *ANEP=len(find(diff(sgn(diff(s)))==±2)+1)/T*. In particular, because the two derivation operations will shorten the length of BCG signal by two sample points, and thus the previous operations cannot identify the extreme point at the penultimate sample point, we utilize the number 1 in the numerator as a compensation, so as to obtain accurate number of extreme points.

*Average Signal Turns Count (ASTC):* Every systole of the heart will generate a shock wave in the blood vessels, and finally form a wave peak in the BCG signal. Likewise, every diastole will lead to a valley. Abnormal heart activities can directly affect the shape of the peaks and valleys. To model the morphological characteristics of the signal peaks and valleys, we introduce a metric named signal turns counts (STC) which has been applied in different research fields and produced impressive results [7]. The main idea of STC is to pick out the signal points that meet certain conditions. Specifically, to adjust to our problem, we define ASTC based on STC as follows:

(7) $$\mathrm{ASTC}=\frac{len\left(find\left(S_{i}\;meet\;condition\;6\right)\right)}{T}$$

(8) $$\{\begin{array}{l} \left[S_{i}-S_{i-1}\right]\times \left[S_{i+1}-S_{i}\right]<0\;\\ \left|S_{i+1}-S_{i}\right|\geq Th,\left|S_{i}-S_{i-1}\right|\geq Th\\ 2\leq i\leq N-1\; \end{array}$$

where *Th* is a threshold which is empirically set as 0.01 based on experimental results. In other words, ASTC counts the sample points that is not only a signal turn in itself, but also has a gap larger than the given threshold *Th* with its two adjacent sample points in terms of amplitude.

#### 3.4.3. Feature Selection

In total, 14 features including 10 HRV-related features and four BCG fluctuation-related features are extracted. Since the proposed hypertension identification method is based on the exploitation of the association relationship among features, one strength of the proposed method is that it is not sensitive to the number of features [28,39]. In other words, it can automatically ignore features that are not helpful for improving classification performance with no requirement for additional feature selection procedure. However, we do need to conduct feature selection for baseline methods, for the sake of fair comparison. In this study, two-sample *t*-test is utilized to select the most useful features. It is a well-known statistical method that is usually employed to test whether there is a significant difference between two sample groups [40]. If the result of the *t*-test, i.e., the *p*-value, is smaller than a specified threshold, we then have reasons to believe that the two groups of samples come from different distributions, in other words, the corresponding feature has excellent discrimination ability. The details of the feature selection process are elaborated in Section 4.2.1.

### 3.5. Hypertension Classification

In this study, we discriminate hypertensive patients and normotensive subjects by designing a CAR-Classifier, which integrates association rules mining and classification together. By this method, the association relationship among multi-dimensional features can be fully investigated. Furthermore, it also generates a set of CARs, which are helpful for doctors to analyze patients’ condition in-depth [41,42,43,44,45,46].

Before elaborating the proposed CAR-Classifier [47,48], we formulize some definitions that are relevant to the CAR-Classifier. In CAR-based classification problems, the dataset is usually viewed as a relational table. Let *D* = {*d*_1_, *d*_2_,…, *d_N_*} represent the dataset, which contains *N* instances described by *k* − 1 distinct attributes. Let *Y* = {*y*_1_, *y*_2_,…, *y_M_*} denote a finite set of class labels that has *M* known classes. Each instance belongs to one of the *M* classes. To simplify the narrative and make it easier to understand, we treat the class label as a special attribute, thus, each instance has *k* distinct attributes. Attributes in *D* can be discrete or continuous, while all of them should be preprocessed uniformly before mining CARs. Concretely, for any discrete attribute, all the possible values are mapped to a set of consecutive positive integers. Similarly, for any continuous attribute, its value range is discretized into several intervals which are further mapped to consecutive positive integers. For example, assume that an instance is described by three attributes (i.e., attribute *A*, attribute *B* and class label *C*) which are discretized into three intervals, four intervals and five categories, respectively. In this case, the intervals of A, B and C are mapped with numbers 1 through 3, numbers 1 through 4, and numbers 1 through 5, respectively. Based on this mappings, an instance can be represented as a set of *<attribute, value>* pairs, and each pair is referred as an *item*. Let *I* = {*item*_1_, *item*_2_,…, *item_L_*} denote the set of items in *D*, which contains *L* items. Afterwards, we define *itemSet* as a subset of *I*. Accordingly, an *itemSet* consisting of *k* items is called *k-itemSet*. Particularly, an *itemSet* that contains only one item is called 1-*itemSet*. Moreover, if a *k-itemSet* (*k* ≥ 2) contains just one *item* derived from the class attribute, we refer this *k-itemSet* as a candidate class association rule (CCAR), which can be represented as a form of follows:

(9) ruleItems→y

where *y* denotes the *item* derived from the class attribute, while *ruleItems* represents the rest *items* of the *k-itemSet*. A CCAR is with *support (sup) s* signifies that *s%* of the instances in *D* contain the *ruleItems* and are labeled with *y*. Meanwhile, a CCAR has *confidence* (*conf*) *c* means that *c%* of instances in *D* that contain the *ruleItems* are labeled with *y*. Let *RuleitemsCount* and *CandidateCount* represent the number of instances in *D* that contain the *ruleItems*, and the number of instances in *D* that contain the *ruleItems* and are labeled with *y*, respectively. Thus, the *sup* and *conf* of a given CCAR can be computed as follows:

(10) $$sup=\left(\frac{CandidateCount}{\left|D\right|}\right)*100\%\;$$

(11) $$conf=\left(\frac{CandidateCount}{RuleitemsCount}\right)*100\%\;$$

where |D| is the number of the instances in *D*. Particularly, a CCAR whose *sup* is greater than a user-specified *minimum support (minSup)* is called frequent candidate class association rule (FCCAR), otherwise called infrequent candidate class association rule (ICCAR). Furthermore, if the *conf* of a FCCAR is greater than a user-specified *minimum confidence (minConf)*, we call this FCCAR a class association rule (CAR).

Next, we give a simple example to illustrate the relationship among the CCAR, FCCAR and CAR. Given a CCAR with form of:

(12) {〈A, 1〉, 〈B, 3〉}→〈C, 5〉

where *A* and *B* are attributes, and *C* is the class label. Actually, the {〈A, 1〉, 〈B, 3〉} and 〈C, 5〉 are the *ruleItems* and *y* in Equation 7, respectively. Assume that the *RuleitemsCount* and the *CandidateCount* of the {<*A*, 1>, <*B*, 3>} are 3 and 2 respectively, and the number of instances in *D* is 10. Thus, the *sup* of the CCAR is 2/10 = 20%, and the confidence is 2/3 = 66.7%. If the *minSup* is specified as 10%, we say that this CCAR is a FCCAR since its *sup* is greater than the *minSup*. Furthermore, if the *minConf* is set less than 66.7%, this CCAR is actually a CAR, and hence can be utilized when constructing the CAR-Classifier.

Based on the concepts defined above, the construction of the CAR-Classifier is elaborated as follows. Concretely, building a CAR-Classifier mainly consists of two stages [34,39,49], i.e., the rule generation stage (RG-Stage) and the classifier build stage (CB-Stage). The former aims to mine all CARs, i.e., CCARs that satisfy specific *minSup* and *minConf* requirements, while the latter is to build the CAR-Classifier based on the extracted CARs.

*RG-Stage:* Since the features extracted in Section 3.4 are continuous values, and cannot be directly used to mine CARs [50], we adopt the equal-width strategy to discretize the feature values into some equal-width intervals. Based on the advice given by doctors, each feature is divided into five equal intervals which represent very low, low, medium, high, very high, respectively. Accordingly, we use numbers 1 through 5 to represent these five intervals. In addition, the class attribute consists of two categories, i.e., hypertensive and normotensive, which corresponds to numbers 1 and 2 respectively.

In order to mine CARs, we design a rule generation algorithm based on the well-known Apriori algorithm [46]. It extracts all the CARs by making multiple traversals over the dataset. Concretely, in the 1_th_ traversal, it picks out all the frequent *1-itemSets*. In the *k* + 1_th_ traversal, it first generate frequent (*k* + 1)-*itemSets* based on the set of frequent *k-itemSets* obtained in the *k*_th_ pass. Then it singles out all CCARs from the obtained frequent (*k* + 1)-*itemSets*. Afterwards, the *sup* and *conf* of these CCARs are computed. At the end of the current traversal, CARs are picked out from the obtained CCARs based on the computed *sup* and *conf*, and the user-specified *minSup* and *minConf*. Eventually, the CARs extracted from each traversals conjointly make up the final CAR set. The details of the proposed rule generation algorithm is described in Algorithm 1, where the function *candidateGen()* represents the linkage of two (*k* − 1)-*itemSets* into one *k-itemSets*.

**Algorithm 1**: The Rule Generation Algorithm
**Definition:**
Let *k-itemSet* denote an *itemSet* that contains *k* items.Let *P_k_* denote the set of *k-itemSet* that may be frequent.Let *C_k_* denote the set of *k-itemSet* that has a class label-derived item, whose *sup* and *conf* are denoted as *supCount* and *confValue* respectively.Let F_k_ denote the set of frequent candidate rules.Let *CAR_k_* denote the set of CARs that have k items.

**Input:**
The user-specified support threshold (*minSup*) and confidence threshold (*minConf*).All the frequent 1-items.

**Output:**
The set of the extracted CARs, which is denoted as *RuleSet*.
1,   *F_1_*={frequent *1-items*}2,   ***for***(k=2; *F_k-1_*≠Ø; k++) ***do***3,     *P_k_*=link(*F_k-1_*);4,     *C_k_*={*p∈ P_k_*∣*p* has a class label-derived item};5,     *F_k_*={*c∈C_k_*∣*c.supCount* ≥ *minConf*}6,     *CAR_k_*={*f∈F_k_* ∣ *f.confValue* ≥ *minConf* }7,   ***end***8,   *RuleSet*=∪_k_*CAR_k_*

*CB-Stage:* For the sake of narrative, the set of CARs is thereafter referred to as *RuleSet*. To improve the classification performance, the most powerful CARs should be employed to construct the CAR-Classifier. To this end, we first sort the *RuleSet* in descending order, where the CARs at the front are of higher classification ability than the CARs at the back. When building the CAR-Classifier, the CARs at the front have the priority to be included into the CAR-classifier. Particularly, the sorting method employed in this paper is as follows:

Given two CARs named *r_i_* and *r_j_*, *r_i_* >*r_j_* (also called *r_i_* precedes *r_j_* or *r_i_* has a higher precedence relative to *r_j_*) if:

(1) the *conf* of *r_i_* is larger than that of *r_j_*, or
(2) their *conf* are the same, but the *sup* of *r_i_* is larger than that of *r_j_*, or
(3) both the *conf* and *sup* of *r_i_* and *r_j_* are the same, but *r_i_* has more items than *r_j_*.

Afterwards, we build the CAR-Classifier based on the sorted *RuleSet*. Concretely, for each CAR *r* in sorted *RuleSet*, we first traverse the dataset *D* to figure out whether *r* can match at least one instance (i.e., the discretized *items* derived from the instance can completely cover the antecedent of rule *r*). If so, we mark the rule *r*, and remove the instances that can be classified by *r* from *D*. Until no more instances can be correctly classified, the majority class of the rest instances in *D* is chosen as a default class. Eventually, all the marked CARs and the default class conjointly make up the CAR-Classifier. The construction of CAR-Classifier is described in Algorithm 2. Particularly, when using the CAR-Classifier to classify an unknown instance, the class label of the CAR that firstly matches the instance is viewed as the classification result. If there is no matched CAR, the instance will be labeled with the default class.

**Algorithm 2**: The Classifier Build Algorithm
**Definition:**
Let *RuleSet* denote the set of extracted CARs, and the *i*_th_ CAR in *RuleSet* is denoted as *r_i_*.Let *D* denote the dataset, and the *i*_th_ case in *D* is denoted as *d_i_*.

**Input:**
The *RuleSet*The dataset *D*.

**Output:**
The CAR-Classifier, which is denoted as *C*.The default class, which is denoted as *DClass*.
1,   *RuleSet*=sort(*RuleSet*)2,   ***for***(i=1; i<*RuleSet.size()*; i++) **do**3,     *temp* = Ø;4,     **for**(j=1; j<*D.size()*, j++) **do**5,       ***if***
*d_j_* is correctly classified by *r_i_*
***then***6,         store *d_j_* in temp and mark *r_i_*;7,     ***end***8,     ***if***
*r_i_* is marked ***then***9,       insert *r_i_* at the end of *C*;10,       delete all the cases in *temp* form *D*.11,   ***end***12,   ***if***
*D* ≠ ∅ ***then***13,     *DClass* = the majority class label of the rest instances in *D*;14,   *C*=*C*∪*DClass*;

## 4. Results

### 4.1. Experimental Setup

Since there are no parameters that need to be trained when building the CAR-Classifier, we randomly divide the dataset, i.e., 128 BCG recordings, into training set and test set by a ratio of 2:1, where the stratified sampling technique is employed to avoid possible bias that may influence the experimental results. The training set is comprised of 85 subjects (including 41 hypertensive patients and 44 normotensive subjects), which is utilized to mine the CARs and build the CAR-Classifier. The test set consists of 43 subjects (including 20 hypertensive patients and 23 normotensive subjects), which is utilized to evaluate the classification performance of the CAR-Classifier. The partition of the dataset is shown in Table 3. To get robust evaluation results, ten-fold-cross-validation is employed. Concretely, the partition of dataset is randomly performed ten times and the average result of the ten folds is regarded as the final classification result.

To evaluate the performance of our method, the methods proposed in [5,6], as well as three other commonly used classifiers, i.e., LibSVM, Decision Tree, and Naive Bayes, are used as the baseline methods. Particularly, the method proposed in [6] extracts HRV-related features from linear domain and nonlinear domain based on ECG, and then directly feeds the extracted features into a LibSVM. The method proposed in [5] first extracts HRV-related feature from multiple temporal resolutions based on ECG signal, and then aggregates them into different feature vectors by using different feature pooling techniques, and the feature vectors are finally fed into L1-regularized logistic regression. For fair comparison, these two baselines are redone based on the dataset collected in this study, with the optimal parameter configurations adopted. On the other hand, the other three baselines utilize the features extracted in this study to identify hypertension, aiming to evaluate the effectiveness of our feature extraction method.

Accuracy (ACC), precision (PRE) and recall (REC) are used as evaluation metrics, which are defined as follows:

(13) $$\mathrm{ACC}=\frac{TP+TN}{TP+FN+TN+FP},$$

(14) $$\mathrm{PRE}=\frac{TP}{TP+FP},$$

(15) $$\mathrm{REC}=\frac{TP}{TP+FN},$$

where *TP*, *TP*, *FP* and *FN* represent true positive, true negative, false positive and false negative, respectively. Specifically, ACC is the fraction of subjects that are correctly classified by the CAR-Classifier. PRE assess how well our classifier is at correctly identifying hypertensive patients, while REC measures the capability of our classifier of not missing hypertensive patients. Higher PRE indicates that fewer hypertensive patients are misdiagnosed, and higher REC signifies that fewer hypertensive patients are missed. Apparently, a good hypertension identification model should achieve high ACC, PRE and REC simultaneously. The experiments are conducted in Matlab 2014a and Eclipse Neon on an Intel Core i5-4590 PC with 8 GB RAM running the Windows 7 operating system.

### 4.2. Evaluation Results

#### 4.2.1. Feature Comparison and Selection

**Figure 5:**
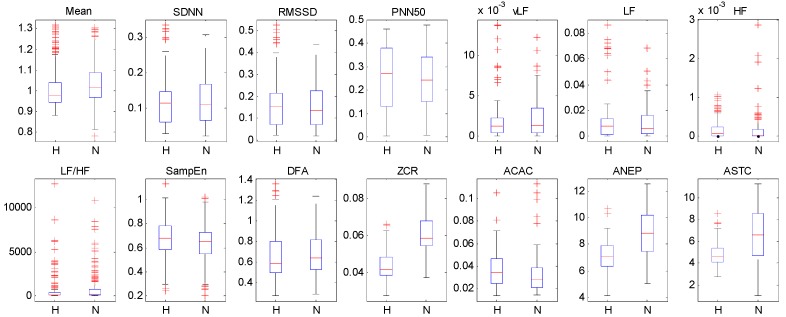
The box diagrams of the extracted features. “H” and “N” represent hypertensive patients and normotensive subjects, respectively.

All the 14 features extracted in this study are summarized in Table 4. For each of them, the mean value, standard deviation and significant difference level (i.e., *p*-value) are computed. From Table 4 we can obtain the following two observations:

First, all the features extracted from non-linear domain and BCG fluctuation analysis are with a significance level of *p*-value < 0.05, but only two features extracted from time domain and frequency domain are with a *p*-value < 0.05, which indicates that analyzing HRV from non-linear domain and the fluctuation pattern of BCG can provide more useful information for identifying hypertension. Second, the *p*-values of SDNN, RMSSD, PNN50, LF, HF, LF/HF are larger than 0.05, which means that these features have poor distinguishing capability. Furthermore, we also visualize the extracted features by using box diagrams in Figure 5. It shows that most of the feature distributions of hypertensive patients and normotensive subjects are quite different from each other except for SDNN, RMSS, PNN50, LF, HF, LF/HF, which corroborates the observations obtained from the Table 4. Based on the above analysis, we discard the six features whose *p*-value are larger than 0.05 and utilize the rest features as the final feature set for the baseline methods.

#### 4.2.2. The Construction of the CAR-Classifier

The *sup* and *conf* are two crucial parameters for the rule generation algorithm (i.e., the proposed Algorithm 1). For example, if the *sup* is too small, the search space will be dramatically expanded and results in numerous unrepresentative CARs, which not only reduces the classification performance but also increases the computational burden. On the other hand, too small or too big *conf* will lead to under-fitting or over-fitting, respectively. The details of the construction of the CAR-Classifier under different combinations of *sup* and *conf* are shown in Table 5, from which we obtain two findings.

First, the time consumption and the number of extracted CARs mainly depend on the *sup* rather than the *conf*. The time consumption significantly upsurges when the *sup* decreases. It is notable that although the CARs need to be extracted only once, too much time overhead is adverse to upgrading the CAR-Classifier in the future. Moreover, too many CARs also make the construction of the CAR-classifier more complicated. To this end, the time consumption and the number of CARs should be controlled in a reasonable range. Second, the ACC and the number of CARs used to build CAR-Classifier remain unchanged under the same *sup*, which indicates that the CAR-Classifier can pick out the most powerful CARs and is not affected by the *conf*. In addition, the number of CARs contained in the CAR-Classifier is less than 50, which is quite practical for doctors and can be utilized as a query manual. Besides, the ACC remains relatively stable when *sup* and *conf* vary. Based on the above analysis, the *sup* and *conf* are set 0.3 and 0.8, respectively.

#### 4.2.3. Classification Performance Comparison

To evaluate the classification ability of features extracted from different aspects, different feature combinations are used as the feature vector and the results are shown in Table 6. It obvious that the performance is the lowest when only the features extracted from time domain are used. In addition, when combining time domain features and frequency domain features together, the performance barely grow, with only an improvement with 0.8% of ACC, 0.4% of PRE, and 1.6% of REC obtained. By contrast, when introducing non-linear domain features and BCG fluctuation features successively, the classification performance increases much more. Concretely, the inclusion of non-linear features brings 3.9% 3.6% and 4.9% improvement in terms of ACC, PRE and REC, respectively. While the utilization of the BCG fluctuation features contributes 4.7%, 4.7% and 5.0% improvement in terms of ACC, PRE and REC, respectively. These experimental results indicate that extracting features that can reflect the non-linearity of HRV and the fluctuation pattern of BCG is conducive to characterizing hypertension pattern much more comprehensively. Particularly, when combining all the features extracted from different aspects together, the classification performance reaches 84.4% of ACC, 82.5% of PRE and 85.3% of REC respectively, which demonstrates the effectiveness of our CAR-Classifier.

**Figure 6:**
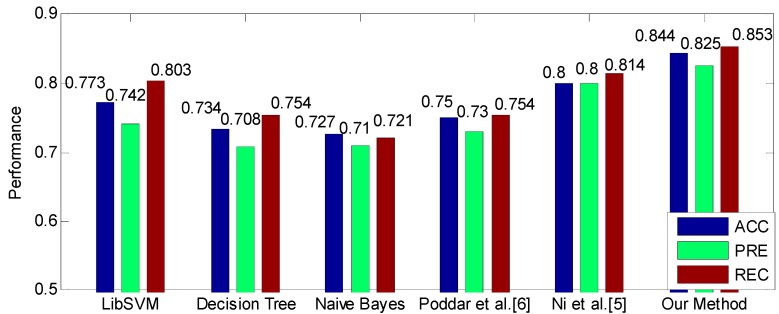
The performance comparison between the proposed method and baseline methods.

The comparison between our method and baselines is shown in Figure 6, which shows that our method achieves the highest performance. Particularly, our method greatly outperforms the method proposed in [6] by 9.4% of ACC, 9.5% of PRE and 9.9% of REC. Compared to the method proposed in [5], our method also improves the performance by 4.4%, 2.5% and 3.9% in terms of ACC, PRE and REC, respectively. In addition, the three common classifiers, i.e., LibSVM, Decision Tree and Naïve Bayes, also obtain acceptable performance with ACC ≥ 72.7%, PRE ≥ 71% and REC ≥ 72.1%. In particular, the LibSVM is the best one among these three classifiers, and achieves 77.3% of ACC, 74.2% of PRE and 80.3% of REC. 

Since the features used in these three classifiers are exactly the features used in CAR-Classifier, therefore, the obtained performance improvement compared to these three classifiers can be attributed to the utilization of CAR-Classifier, which indicates that extracting association relationship among features is conducive to modeling the hypertension pattern more accurately.

#### 4.2.4. The Utility of the CARs

**Figure 7:**
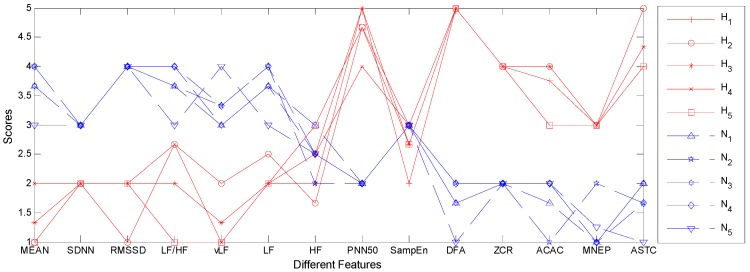
The visualization of the five most powerful CARs in hypertensive group and normotensive group. The null value in CARs are replaced by the average value of current group, and the CARs for hypertensive group and normotensive group are plotted by using red solid line and blue dotted line respectively.

In this section, we evaluate the utility of the extracted CARs in practice. To this end, the first five precedent CARs respectively in hypertensive group and normotensive group are shown in Table 7, from which we can find that the CARs in two groups are significantly different from each other. Specifically, the time domain features and frequency domain features in hypertensive group usually have smaller values compared with that in normotensive group, excepting for the PNN50. By contrast, the non-linear features and BCG fluctuation features in hypertensive group are generally greater than that in normotensive group. These experimental results indicate that the function of the autonomic nervous system degrades and the cardiac activities becomes chaotic for hypertensive patients, which is consistent with existing medical research results [29,32,51]. That is, the extracted CARs do reflect the nature of hypertension and can be used for clinical guidance. In order to understand the extracted CARs more intuitively, we also visualize them in Figure 7, where the null values in Table 6 are replaced with the average value of current group. Obviously, the overall trends of these two sets of CARs are quite different. Particularly, the overall trend of the CARs in hypertensive group remains relatively low at first, but it gradually increases till the end, excepting for the fluctuations at PNN50, SampEn, and DFA. As for the CARs in normotensive group, the overall trend is just the opposite. It almost constantly goes down from beginning to end. Based on the above results, we claim that doctors can analyze patient’s condition more accurately with the help of these CARs. For example, if a subject’s feature values converge towards to or are similar with the CARs in hypertensive group, the doctors have reasons to believe that this subject is likely to suffer from hypertension in the future, and hence effective health interventions should be applied on him or her at once. In this way, the occurrence of high blood pressure or the deterioration of the condition can be prevented effectively.

We also conduct a small-scale user study to evaluate the practical value of the extracted CARs in clinical diagnosis by designing a scoring system. Particularly, this user study focuses on evaluating the CARs form the following aspects: (1) Consistency, the level of consistency between the CAR-based classification results and the clinical diagnosis results (0: Not consistent, 5: Very consistent); (2) Correctness, the extent that these CARs can correctly reflect the physiological status of hypertensive patients and normotensive subjects (0: Completely wrong, 5: Completely correct); (3) Helpfulness, the amount of information these CARs can provide for doctors to analyze the patients’ condition (0: None, 5:Very much); (4) Usability, the degree of difficulty for doctors to master these CARs (0:Very hard, 5: Very easy). Specifically, “not consistent” means that the CAR-based classification results have no relevance to the clinical diagnosis results; “completely wrong” indicates that these CARs completely unable to reflect the subjects’ real physiological status; “none” signifies that doctors can’t obtain any useful information for diagnosing hypertension; and “Very hard” means that the doctors cannot master this technique in a certain period of time. Note that the minimum scoring interval is set to 0.5, aiming to get a tradeoff between accuracy and ease of use.

**Figure 8:**
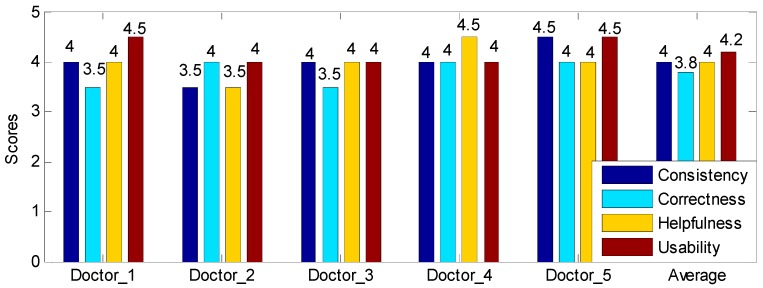
The user study results. The score range is 1 to 5, and the minimum scoring interval is 0.5.

In this study, a total of five cardio-vascular doctors are recruited, with each of them has at least ten years of clinical experience. The scores given by each doctor are shown in Figure 8, from which we obtain two observations. First, all five doctors give high scores for these four criteria. Particularly, all the scores are higher than 3.5, and most of them are higher than or equal to 4.0, which means that five doctors unanimously endorse these CARs. In addition, the average scores of these four criteria are 4.0, 3.8, 4, and 4.2, which demonstrates the validity of the extracted CARs. Second, the variance of the four criteria are 0.125, 0.075, 0.125 and 0.075, respectively, which indicates that five doctors have similar views on these CARs. 

In other words, the extracted CARs show relatively stable performance for different people. The results of this user study show that the proposed CAR-based hypertension identification method can assist doctors to diagnose hypertension in clinical to some extent, and hence has a certain promotion value.

## 5. Discussion

Hypertension is an important cause of chronic health problems globally. Early and accurate identification of hypertension is important for better prognosis and preventing the progression of the disease [1]. In this paper, we propose a CAR-based hypertension identification method based on the utilization of the association relationship among multi-dimension features. Particularly, it integrates classification and association rule mining together, and hence makes it possible to obtain the CARs that can reflect the subjects’ physiological state. The proposed method is proved to be able to improve the diagnosis accuracy.

The mining of CARs relies on the association relationship among features, thus the process of feature extraction plays an important role in improving the hypertension identification performance. In this paper, we not only extract HRV-related features from time domain, frequency domain, and non-linear domain, but also extract features to model the fluctuation pattern of BCG. Experimental results show that extracting features from multiple aspects characterizes hypertension pattern more comprehensively, and hence generates more informative CARs for classification. On the other hand, the construction of the CAR-Classifier is insensitive to the number of features. Features that are irrelevant to the final model will be automatically ignored without requirement for an additional feature selection procedure. However, it doesn’t imply that we can extract features as much as possible, since too much features will significantly increase the time overhead and computational burden. Moreover, extracting too many features also results in numerous CARs, which complicates the process of the construction of the CAR-Classifier. In addition, the common feature selection methods like Information Gain (IG) treat each feature as independent individuals [52], which tends to destroy the association relationship among features and hence results in useless CARs. Therefore, specialized feature selection techniques should be considered if the number of features is too large.

Most classifiers such as SVM only report the classification results. However, these results are usually unexplainable, making it difficult to take full advantage of these results. Some other classifiers such as C4.5, can generate a set of IF-THEN rules along with the classification results. However, these rules can only explain why such classification results are produced to some extent. Moreover, the C4.5 considers each feature individually when constructing the decision tree, instead of considering them simultaneously, which may leave out some valuable association relationship among features. Furthermore, the rules generated by C4.5 rely heavily on the values of the continuous attributes. Even if the dataset changes slightly, the structure of the decision tree, especially the nodes representing continuous attributes, is likely to change, and hence leads to the change of the generated rules. On the other hand, the CAR-Classifier deems the extracted features non-independent, which is more in line with the actual situation. Therefore, it can reflect hypertension pattern more accurately. Additionally, since the continuous attributes have been binned into several intervals, the mining of the CARs does not depend on the attribute values too much. Slight changes of the dataset will not influence the extracted CARs, which shows the robustness of our method. Precisely because of this, the proposed method is suitable for scenarios where the data quality is poor, for example, wearable or non-intrusive devices based health monitoring tasks [53].

An important advantage of the proposed method is that it can generate a set of useful CARs. Experimental results show that these CARs are capable of reflecting the subjects’ physiological status. In particular, hypertensive patients tend to have smaller time-frequency domain features but larger non-linear features and more fluctuant BCG signal, which is just the opposite of the normotensive subjects. In addition, we can easily obtain more meaningful information from the visualized CARs. For instance, if someone’s feature curve does not coincide with hypertensive pattern or normotensive pattern but tends to one of them, we can believe that the subject’s physiological state is closed to the pattern represented by the closer curve. Particularly, the results of the user study indicate that the extracted CARs have the potential to be used as assistive tool for doctors in diagnosing hypertension. Furthermore, the extracted CARs can also be applied to daily health monitoring services. For example, once it is detected that the user’s physiological state is changing from normotensive to hypertensive, the user should be alarmed and takes effective medical interventions immediately, which is because that the user may be at risk of suffering from hypertension and even have serious sub-clinical effects of hypertension already [54].

The limitation of the proposed method is that the smart mattress-based BCG collection process limits user’s active boundary and is hard to obtain high-quality signals. In other words, users must be on the bed when collecting BCG signals, which cannot investigate their physiological state when they perform daily activities. To tackle this problem, other forms of equipment should be included. For example, the smart watch [25], wearable devices [55] and smart chair [26] are good ways to collect physiological signals in daytime. Particularly, the PPG based non-invasive blood pressure estimator [21] is a promising technique since it can estimate blood pressure continuously and accurately. In addition, other signal preprocessing methods should be employed [56,57,58,59]. By combining the smart mattress proposed in this study and other emerging devices and signal preprocessing methods, the pattern of hypertension can be characterized more comprehensively.

## 6. Conclusions

In this paper, we present a novel hypertension identification method based on the integration of classification and association rule mining, which is able to transform the association relationship among features into useful CARs, and hence to identify hypertension accurately. In particular, we extract features from multiple aspects, including time domain, frequency domain, non-linear domain and especially the BCG fluctuation features, to characterize hypertension pattern more accurately. In addition, we select the most powerful CARs to build the CAR-Classifier. These two points are the key to improving the hypertension identification performance. Experimental results based on a real dataset consisting of 128 subjects show that the proposed method achieves 84.4%, 82.5% and 85.3% in terms of ACC, PRE, and REC respectively, which significantly outperforms the baseline methods. Moreover, the byproducts of our approach, i.e., the extracted CARs, are proved to be able to reflect the subjects’ philological status, which indicates that it can be used as assistive tools for doctors to analyze the subject’s condition. More than that, the results of the user study based on five clinicians demonstrate the high utility of these CARs, which shows the promotion value of our method. In the future, we will focus on building a classifier that needs fewer CARs but achieves higher identification performance. In addition, we will also include other physiological signals especially the PPG [21], the ECG [60,61,62,63], and the EEG [64], in order to get a better understanding of the patient’s condition.

## Figures and Tables

**Table 1:** Statistics of the experimental BCG dataset (mean ± standard deviation).

Subject Information	Hypertensive	Normotensive
Number	61	67
Sex (Male/Female)	33/38	35/32
Age (years)	55.6 ± 7.9	53.2 ± 9.2
Heart Rate (bpm)	77.1 ± 9.2	73.6 ± 8.3
Body Mass Index (kg/m^2^)	24.3 ± 3.6	23.7 ± 3.3
Systolic blood pressure (mmHg)	155.6 ± 11.2	112.1 ± 15.7
Diastolic Blood Pressure (mmHg)	103.6 ± 8.2	74.4 ± 6.3

**Table 2:** List of the extracted features.

Type	Features	Description
TD ^1^	Mean	The mean value of RR intervals
	SDNN	The standard of successive RR intervals
	RMSSD	The root mean square of successive RR intervals
	PNN50	The percentage of RR intervals longer than 50ms
FD ^2^	vLF	The power in 0.0033 Hz–0.04 Hz band
	LF	The power in 0.04 Hz–0.15 Hz band
	HF	The power in 0.15 Hz–0.4 Hz band
	LF/HF	The ratio of power in LF and HF band
ND ^3^	SampEn	The sample value with r = 0.15 * STD
	DFA	The short-term coefficient of detrended fluctuation analysis
BF ^4^	ZCR	The zero crossing rate of BCG signal
	ACAC	The average cumulative amplitude change in unit length
	ANEP	The average number of extreme points in unit time
	ASTC	The average signal turn counts in unit time

^1^ Time domain features. ^2^ Frequency domain features. ^3^ Non-linear domain features. ^4^ BCG fluctuation features.

**Table 3:** The division of the experimental BCG dataset.

Group	Sex	Number	Training Set	Test Set	Ratio ^1^
Hypertensive	Male	33	22	11	66.7%
	Female	28	19	9	67.9%
Normotensive	Male	35	23	12	65.7%
	Female	32	21	11	65.6%
Total	-	128	85	43	66.4%

^1^ The ratio of the training set to the test set.

**Table 4:** A summary of the extracted features (mean ± standard deviation).

Type	Features	Hypertensive	Normotensive	*p*-Value
TD ^1^	Mean	1.01 ± 0.10	1.04 ± 0.10	0.0082
	SDNN	0.11 ± 0.06	0.12 ± 0.06	0.2653
	RMSSD	0.15 ± 0.10	0.15 ± 0.09	0.6766
	PNN50	0.25 ± 0.14	0.25 ± 0.14	0.4171
FD ^2^	vLF	0.002-0.002	0.006 ± 0.004	0.0178
	LF	0.01-0.013	0.013 ± 0.01	0.7804
	HF	0.05 ± 0.01	0.04 ± 0.01	0.5354
	LF/HF	591.6 ± 1445	762.1 ± 1477	0.1803
ND ^3^	SampEn	0.68 ± 0.15	0.63 ± 0.14	0.0027
	DFA	0.65 ± 0.23	0.70 ± 0.20	0.0438
BF ^4^	ZCR	0.05 ± 0.01	0.05 ± 0.01	0.0001
	ACAC	0.11 ± 0.03	0.14 ± 0.06	0.0247
	ANEP	8.74 ± 1.76	6.97 ± 1.31	1.7 × ^−7^
	ASTC	6.68 ± 2.71	4.88 ± 1.20	1.2 × ^−6^

^1^ Time domain features. ^2^ Frequency domain features. ^3^ Non-linear domain features. ^4^ BCG fluctuation features.

**Table 5:** The construction of the CAR-Classifier using different support and confidence.

Sup ^1^	Conf ^2^	Time Overhead(s)	Number of Extracted CARs	Number of CARs Used in CAR-Classifier	ACC (%)
0.3	0.80	251.8	7016	38	84.4
	0.75	249.2	8580	38	84.4
	0.70	255.7	8616	38	84.4
0.25	0.80	1914.9	37,848	44	84.7
	0.75	1909.8	55,780	44	84.7
	0.70	1912.3	55,852	44	84.7
0.2	0.80	6710.1	74,460	48	83.2
	0.75	6663.4	104,136	48	83.2
	0.70	6599.5	104,244	48	83.2

^1^ The support threshold. ^2^ The confidence threshold.

**Table 6:** The most powerful CARs in hypertensive group and normal group.

Feature Combination	ACC (%)	PRE (%)	REC (%)
TD ^1^	75.0	73.8	73.8
TD+FD ^2^	75.8	74.2	75.4
TD+FD+ND ^3^	79.7	77.8	80.3
TD+FD+ND+BF ^4^	84.4	82.5	85.3

^1^ Time domain features. ^2^ Frequency domain features. ^3^ Non-linear domain features. ^4^ BCG fluctuation features.

**Table 7:** The most powerful CARs in hypertensive group and normotensive group.

Type	Features	Hypertensive Group ^5^					Normotensive Group				
		CAR 1	CAR 2	CAR 3	CAR 4	CAR 5	CAR 1	CAR 2	CAR 3	CAR 4	CAR 5
TD ^1^	Mean	-	1	-	2	1	-	4	-	4	3
	SDNN	-	2	2	-	2	3	3	-	-	3
	RMSSD	2	1	2	2	2	4	-	4	4	-
	PNN50	5	-	5	4	-	-	-	2	2	2
FD ^2^	vLF	-	2	-	1	1	3	-	3	-	4
	LF	2	-	-	2	2	-	4	-	4	3
	HF	-	-	2	-	3	3	2	-	-	-
	LF/HF	2	-	2	-	1	-	-	4	4	3
ND ^3^	SampEn	2	3	-	3	-	3	-	-	3	-
	DFA	-	-	5	5	5	-	2	-	2	1
BF ^4^	ZCR	4	4	-	-	-	2	-	2	-	-
	ACAC	-	4	4	4	3	-	1	-	2	2
	MNEP	3	-	3	-	3	1	2	1	1	-
	ASTC	4	5	-	-	4	2	-	2	-	1

^1^ Time domain features. ^2^ Frequency domain features. ^3^ Non-linear domain features. ^4^ BCG fluctuation features. ^5^ 1 to 5 and symbol “-” represent “very low”, “low”, “medium”, “high” “very high”, and “null”, respectively.

## Supplementary Materials

The developed software code and the collected BCG dataset are available online at https://doi.org/10.6084/m9.figshare.7594433.
